# Business and public health collaboration for emergency preparedness in Georgia: a case study

**DOI:** 10.1186/1471-2458-6-285

**Published:** 2006-11-20

**Authors:** James W Buehler, Ellen A Whitney, Ruth L Berkelman

**Affiliations:** 1 Center for Public Health Preparedness & Research and Department of Epidemiology, Rollins School of Public Health, Emory University, Atlanta, Georgia, USA

## Abstract

**Background:**

Governments may be overwhelmed by a large-scale public health emergency, such as a massive bioterrorist attack or natural disaster, requiring collaboration with businesses and other community partners to respond effectively. In Georgia, public health officials and members of the Business Executives for National Security have successfully collaborated to develop and test procedures for dispensing medications from the Strategic National Stockpile. Lessons learned from this collaboration should be useful to other public health and business leaders interested in developing similar partnerships.

**Methods:**

The authors conducted a case study based on interviews with 26 government, business, and academic participants in this collaboration.

**Results:**

The partnership is based on shared objectives to protect public health and assure community cohesion in the wake of a large-scale disaster, on the recognition that acting alone neither public health agencies nor businesses are likely to manage such a response successfully, and on the realization that business and community continuity are intertwined. The partnership has required participants to acknowledge and address multiple challenges, including differences in business and government cultures and operational constraints, such as concerns about the confidentiality of shared information, liability, and the limits of volunteerism. The partnership has been facilitated by a business model based on defining shared objectives, identifying mutual needs and vulnerabilities, developing carefully-defined projects, and evaluating proposed project methods through exercise testing. Through collaborative engagement in progressively more complex projects, increasing trust and understanding have enabled the partners to make significant progress in addressing these challenges.

**Conclusion:**

As a result of this partnership, essential relationships have been established, substantial private resources and capabilities have been engaged in government preparedness programs, and a model for collaborative, emergency mass dispensing of pharmaceuticals has been developed, tested, and slated for expansion. The lessons learned from this collaboration in Georgia should be considered by other government and business leaders seeking to develop similar partnerships.

## Background

State and local health departments throughout the United States are working to improve their ability to address the public health consequences of bioterrorism. The federal Centers for Disease Control and Prevention (CDC) and the Health Resources Services Administration (HRSA) are supporting these efforts, which are increasingly geared to preparing for a spectrum of public health emergencies, including different forms of terrorism, infectious disease epidemics such as pandemic influenza, and natural or environmental disasters[1-5]. Federal support includes both funding and technical assistance.

Even with increased federal support, state and local governments will be unlikely to adequately manage a large-scale emergency response alone and must involve community partners, including businesses. As was evident from Hurricane Katrina in 2005, businesses can provide essential resources to supplement government efforts [6,7]. These resources include supplies and inventories, storage facilities, distribution systems, vehicles, communications links, and people with a mix of expertise and skills. While ad hoc engagement of businesses during a crisis can be invaluable, business involvement should preferably be based on prior collaboration with government agencies to develop and test emergency plans, including delineation of roles, responsibilities, and procedures for various components of response and recovery.

Following the anthrax attacks of 2001, the National Business Group on Health surveyed selected large companies and public health leaders regarding their experience with collaboration. Survey responses revealed that opportunities for business and public health partnerships were underdeveloped, in large part because personal relationships between business and public health leaders were limited. As a result, business and public health leaders lacked a working understanding of one another's capabilities and vulnerabilities and, thus, of optimal ways to engage in mutually beneficial collaborations [8,9].

The distribution and dispensing of supplies from the Strategic National Stockpile (SNS) is a good example of an opportunity for business-public health partnerships. The SNS is a cache of emergency medications and supplies maintained by CDC, and state and local governments are responsible for receiving, distributing, and dispensing SNS supplies [10]. Under the Cities Readiness Initiative, the core planning scenario is a hypothetical, massive exposure to aerosolized anthrax [11]. CDC has charged state and local health departments with developing and testing procedures to administer post-exposure antibiotic prophylaxis to the population of a large urban area within 48 hours of an attack. This task presents a complex mix of logistic, medical, and communication challenges, exactly the kind of effort that could benefit from the combination of government and business human and material assets.

In Georgia, public health officials and business leaders have been working for several years to develop a collaborative SNS dispensing model. Initially, the partnership involved the state's Division of Public Health, two local public health districts in the metropolitan Atlanta region, and the Georgia Business Force, an integral part of the Metro Atlanta Region of the Business Executives for National Security (BENS). As a national organization with offices in multiple regions of the country, BENS provides a "channel through which senior business executives can help enhance the nation's security" [12]. The purpose of its Business Force program is to build public-private partnerships at state or regional levels "to help close gaps in homeland security that neither government, nor business, can fill alone"[13]. BENS is a non-partisan, not-for-profit 501(c)(3) tax-exempt organization [14], which is underwritten primarily by individual and corporate membership fees and corporate donations, and the Georgia Business Force is an alliance of businesses within the Atlanta Metro Region of BENS. For brevity, we will use the abbreviation "BENS" to refer interchangeably to the Metro Atlanta Region of BENS and its Georgia Businesses Force. The SNS dispensing project is part of broader BENS collaborations with public health, public safety, and homeland security agencies in Georgia aimed at enhancing all hazards emergency preparedness. The SNS project grew from contacts between BENS and public health leaders that had been established through prior collaboration on state homeland security committees and in developing a primer for businesses on bioterrorism and emergency preparedness [15].

In July 2005, the Georgia partners tested their SNS dispensing model in exercises at three sites, including two school-based sites managed by local public health officials and one company-based site managed primarily by business volunteers. BENS members recruited approximately 1,200 volunteers from their companies to serve as exercise evaluators and mock patients. The exercise successfully stressed procedures for staging the sites and mass dispensing, identified strengths and weaknesses in the dispensing model, and led to substantial revisions in plans for future dispensing. At present (November 2006), planning is underway to involve company-based and public health-managed distribution sites in all five health districts in metropolitan Atlanta in further SNS dispensing exercises (for additional information on the history of the BENS-public health partnership in Georgia, see Additional File 1). In this model, company-managed dispensing sites will serve company employees and family members; and, when dispensing is complete at company facilities, company employees will serve as volunteer staff at public health sites. For governments, envisioned benefits include delivery of services to large numbers of people through the company-managed dispensing sites, an accompanying reduction in demand for services at public health sites, and the availability of company volunteers to serve as volunteer staff at public health sites. For companies, the envisioned benefit includes access to medications for their employees and families, as well as the opportunity to support community response efforts.

The partnership between BENS and public health in Georgia exemplifies both the opportunities and challenges of collaboration between businesses and public health agencies. This report summarizes findings from an investigation that aimed to identify lessons learned from the BENS-public health partnership in Georgia and to inform the development of similar partnerships elsewhere.

## Methods

We conducted a case study based on the "success case method" described by Brinkerhoff [16]. This method is geared for use in business and government settings to evaluate new projects, illustrate accomplishments, and identify best practices. We interviewed current or former local and state government officials, BENS staff and members, academicians, and representatives from CDC, all of whom have been involved in the BENS-public health partnership in Georgia. Although our focus was on public health, interviews with government officials included representatives of public safety and homeland security agencies in order to describe the context of the BENS-public health partnership in Georgia.

We developed the study objectives, questionnaire, and list of interviewees in collaboration with representatives of the Georgia Division of Public Health and BENS (see Acknowledgements). We identified 26 people we wished to interview, all of whom agreed to participate and completed an interview with project staff (see Additional file 2 for a listing of persons interviewed).

The interview consisted of 11 open-ended questions (Additional file 3) and allowed for follow-up questions, clarifications, or probes. Questions addressed the respondents' motivation for participation in the partnership, the process for establishing priorities and selecting projects, barriers and facilitators that affected the collaboration, the benefits of the partnership, the limits of what such partnerships can achieve, and recommendations.

Interviews were conducted by either the Principal Investigator (JB) or the lead Co-Investigator (EW). For 10 of the 26 interviews, both interviewers were present with one conducting the interview and the other serving as an observer, especially at the start of the project, to improve consistency of the interview process. With the exception of two interviews that were conducted by telephone, all interviews were conducted in person and took 30–60 minutes. The interviewers took handwritten notes and reviewed and amended their notes after each interview, usually within 24 hours. The interviewers conferred and reviewed one another's notes regularly to keep abreast of themes and key points that emerged; upon completion of the interviews, they reviewed the notes to identify themes and illustrative quotations. Respondent quotes (italicized in the text) are taken from the interview notes.

Respondents provided verbal informed consent to participate in the study. The study protocol was reviewed and approved by the Emory University Institutional Review Board.

## Results

### Motivation for participation

Many BENS members have had prior careers in the military, and they value the opportunity that BENS offers to "continue to serve." The motivation of BENS members is broadly shaped by the realization that governments and business each have resources that, if combined, could enhance emergency preparedness for the benefit of governments, communities, and businesses. Business respondents appreciate that public health officials have expert knowledge about biological and other health threats, unique access to information and resources in crisis situations, and skills and authorities essential for responding to public health emergencies. BENS members emphasize that their organization is geared to providing community service, not to advancing short-term business interests. The experience of one member is typical of the motivation of business participants:

"...I looked at opportunities and ways I could get involved...I heard about BENS and saw that this was one where I could help lead and make some change...I attended a conference in Washington DC about the response to 9/11. There was a presentation on BENS and I got hooked...When I got home a few days later I called and asked 'How can I get involved?'"

While the principle of community service is fundamental to BENS, many members readily acknowledge that their participation is not purely altruistic. In addition to the good publicity that can accompany community service, many view their involvement as an extension of business continuity planning. The survival of businesses during and following a disaster depends on the survival of communities and vice versa. Business respondents were keenly aware of the potential for government systems to be overwhelmed and the attendant consequences. "*Fear of failure and a clear understanding of the limits of government*" combined with a sense that "W*e're a part of the community and should be a part of the solution*" were perspectives that exemplify the motivation of BENS members.

From the perspective of public health agencies, the motivation to work with businesses is simple. Public health officials recognize their limitations and see the value of business resources for emergency preparedness and response. With respect to SNS dispensing, this includes logistics expertise, access to large numbers of potential volunteers, and business infrastructures for training, organizing, and communicating with volunteers. The motivation for public health to work with an organization such as BENS also reflects a desire for simplicity because BENS has ties to multiple businesses, reducing the need for public health to cultivate links independently with different businesses. BENS represents many of the largest companies in metropolitan Atlanta, as well as smaller firms with specific expertise relevant to emergency preparedness. The ability to reach these companies through BENS is clearly valuable to public health, given both the assets these companies can enlist in an emergency and the number of people they can reach. As one respondent observed, "*We are surrounded by opportunities*."

### Challenges to collaboration

Differences between the cultures of business and government in general and public health in particular represented the most frequently cited challenge to collaboration. Manifestations of these differences included a lack of familiarity with one another's values, metrics, resources, constraints, lines of accountability, management styles, lingo, and modes of operation. As a result, business leaders and public health officials often do not know how to approach one another or whom to call to establish a relationship.

Additionally, there is a stereotypical view that people in business and public health have of one another. Many in public health are suspicious of the profit motive of businesses and are thus guarded when approached by business representatives. Entrees from business representatives may be viewed with suspicion, e.g., "W*hat is this guy trying to sell me?*" This perspective was amplified after September 11, 2001 and the subsequent anthrax attacks, when government officials felt besieged by entrepreneurs seeking to promote new security or health protection products. From the business perspective, suspicion of government reflects a perception that government officials typically want "*something for nothing," *often seek to impose burdensome regulations, and have a bureaucratic mindset. As one BENS staff member noted:

"We need to get past the image of the public employee as someone resistant to change, just works 9-5...couldn't get a real job"

There is also a perception among some business executives that government management styles are at times inefficient and wasteful. This latter concern is a manifestation of differences in the ways that business and government value efficiency and effectiveness. While both are important to businesses and governments, one business respondent characterized the difference in the following way.

"*In business, you are constantly trying to shift dollars from the cost to the profit side of the ledger. The way you do that is to improve efficiency. If something costs you a dollar, the first question a good manager asks is whether you can get that cost down to ninety-five cents. When you get it down to ninety-five cents, the next question is how to get it down to eighty-seven point five. If you don't do that, your competitors will do it for you, and the next thing you know you're out of business. In government, the priority is making sure you can get the job done [effectively], and it is less important that you do it at the lowest possible cost. That's why...when public health approaches business and asks for 200 volunteers for an exercise, the first reaction from someone in business person is likely to be, 'Why wouldn't 100 volunteers be enough?*"'

This difference, viewed from the perspective of a public health official was described in the following way:

"Public health and business people speak a different language. Business people...focus on measurable outcomes based on dollars. Public health people are [concerned about the] well-being of humanity, but you can't reduce that to dollars..."

Historically, business-government interactions have often centered on regulation or investigations that may lead to regulation, casting business and public health people into adversarial positions. From this perspective, business people are apt to worry about misinformed or unnecessary government regulation, while people in public health may see their role as constraining businesses from promoting unhealthy products, polluting the environment, or threatening the health of workers. This perspective has also blinded many in public health from recognizing the critical role of businesses in assuring community continuity. The notion that perceptions of business are shaped by adversarial engagements with governments is troubling to BENS members:

"*Most business leaders cringe when tobacco is held up as an example of business in public health...You only hear about business when there's fraud. Our members are the first to argue that bad actors [should] get what they deserve*."

As cultural gaps have narrowed through the establishment of trust and collaborative efforts to act in response to shared objectives, other issues have become apparent. These include concerns about the following:

#### Government procurement regulations

Business leaders perceive that procurement regulations force government officials to maintain a defensive posture in order to avoid risking a future conflict of interest, or appearance of conflict of interest. Both may be concerned that collaboration could poison opportunities for future contractual interactions, especially if a business partner is perceived as having unfair insight into government contracting opportunities as a result of voluntary collaborations.

#### Potential for shifts in government priorities

At times, business partners were frustrated by shifts in public health priorities, which some BENS members described as a "*flavor of the month*" mentality. For example, over the course of the SNS project, public health and other government agencies in Georgia had to divert part or all of their attention to the Group of Eight (G8) summit conference in June 2004, pandemic influenza planning, and other preparedness mandates from CDC and HRSA. While these diversions are understandable, many business leaders have a "*Lets get it done*" attitude, they value adherence to timelines, and they are concerned when competing demands on public health officials force project delays. People who work in government are familiar with such political realities and have at times been frustrated by the impatience of business partners. In addition, many in business have been surprised to learn that state and local public health agencies lack sufficient staff and resources to manage their multiple preparedness responsibilities simultaneously. As one public health official noted:

"It was hard for business leaders to even imagine that public health was not well funded and did not have the tools we need given our responsibilities...They think we have more money than we do."

Anxiety about the timeline for exercises was felt most acutely by some in local public health departments:

"*BENS wants to do more exercises. We're exercised to death. They don't understand how much work it is for us to put on an exercise*."

"*It's too soon to have another exercise...we needed more time to train...These timelines are crazy. We have other exercises and other responsibilities*"

Business respondents had a different reaction to delays, but also demonstrated a growing understanding of the constraints affecting their public health partners:

"*If planning for SNS is delayed, business partners ask, 'What if we have an anthrax attack? If we postpone the exercise then we're postponing our ability to be prepared*...'"

"*Putting on my military hat, you don't back up or postpone. You make it happen. The tendency of public health to shift priorities and postpone projects is frustrating...But public health said 'Slow down' and BENS has to understand that...At least we're moving forward*."

#### Different management styles

Business leaders observed that company chief executive officers often exercise greater authority than government officials to mandate changes or enforce adherence to policies and standards across locations. Public health managers put greater emphasis on consensus-based decision making, and they value flexibility in adapting programs to local circumstances. This partly reflects the sharing of public health authority in Georgia between the state health officer (the Director of the Division of Public Health) and directors of the state's 18 local public health districts. As a consequence, public health districts in Georgia are apt to develop SNS dispensing procedures that are more independent and variable than a company's operations in different locations.

#### Occasional but memorable instances of government disorganization

The state's experience in responding to Hurricane Katrina, which directly affected nearby states and resulted in evacuation of affected people to Georgia, was largely viewed as an example of the benefits of positive links between business and government agencies. There were, however, instances when government officials asked BENS staff to request resources from members, and business leaders responded quickly, only to be told that needs had shifted and their resources were not needed. In another instance, a Georgia BENS member offered to provide a critical service at no cost to the Federal Emergency Management Agency (FEMA), but FEMA did not have an administrative mechanism to accept the offer. While this episode reflected an interaction with a federal rather than Georgia agency, the perceived lack of flexibility or imagination by a government agency unavoidably shaded perceptions of governments in general, including state government, among some business leaders.

#### Confidentiality of proprietary information

Concerns about the confidentiality of information that businesses share with governments and vice versa have been an ongoing challenge, despite progress in clarifying laws and regulations that govern information sharing and access. Much of the critical infrastructure required to support community functioning is privately owned. Maintaining an inventory of these assets is essential to emergency planning, but information about these resources may be highly sensitive for either security or proprietary reasons. Businesses' enthusiasm for sharing such information with government agencies in advance of crises is diminished if it is susceptible to widespread access via open record laws. Likewise, government agencies may acquire intelligence that would be valuable to businesses in protecting assets, and businesses may be uniquely positioned to assist governments in interpreting intelligence reports, especially if they relate to potential threats against businesses. Despite the potential benefit of sharing intelligence information, government officials may feel compelled to withhold it, erring on the side of making sure they adhere to security regulations as they understand them. The status of efforts to address these concerns was summarized by one government respondent in the following way:

"The current mindset is 'Hold onto your information'...We're chipping away at that..."

#### Liability

Another major concern for businesses is liability, especially with regard to collaboration in exercises. For example, if a company offers space to conduct an exercise or allows its employees to serve as volunteers, a host of questions arise about potential liability in the event of an injury. While Good Samaritan laws typically protect people engaged in supporting an emergency response, they generally do not protect preparedness activities, nor do they protect companies. Moreover, the level of protection may be narrowly defined by the specifics of an emergency declaration. Both liability and confidentiality concerns tend to evaporate during an actual crisis, but, in the absence of advance planning, governments have been unprepared and unable to accept valuable offers for help during crises. Ironically, current open record and Good Samaritan laws deter some business from engaging in such pre-event planning.

#### Ongoing differences in perspective

Despite successes in bridging cultures, managing differences in perspective remains an ongoing challenge. For example, the July 2005 SNS dispensing exercise was widely viewed as successful by business and public health respondents, both for providing proof-of-concept for the collaborative dispensing model and for evaluating specific procedures. Despite this shared view, several public health respondents expressed frustration because BENS member companies did not meet initial targets for the number of volunteers they could recruit:

"*I had to do a lot of volunteer recruiting that I had expected them [BENS] to deliver*..."

"S*how me the volunteers*."

For others, this shortfall was immaterial, as exemplified by the following observation from a business respondent:

*"What is the question? Is the question Can we get the number of targeted volunteers to an exercise?... Or is it can you learn a lot real quick?*... *We set a goal of 2000 volunteers and got 1200. I don't see that as a failure. The exercise truly flexed the process...We had enough to truly stress the operation and learn where the flaws were in the design of the operation*..."

While this inventory of challenges may seem daunting, none of these obstacles was viewed as insurmountable, nor were they viewed as a deterrent to planned expansions of the SNS dispensing model or extension of the partnership to pandemic influenza planning. To the contrary, respondents emphasized that their growing understanding of one another's concerns and their increased ability to be frank with one another about these issues were important signs of progress. Identifying potential barriers to growth in collaboration has enabled the partners to target these concerns, including ongoing work to address legal questions surrounding confidentiality and liability.

### Facilitators to collaboration

The ability of business and government partners to establish personal relationships has been fundamental to the success of the partnership. The accompanying growth in trust and respect and the ability of participants to enjoy working together have enabled the partnership to take on increasingly complex activities. As one business respondent observed at the conclusion of the interview, "*It's just been fun*" and as one state official observed:

"They're good people. Their hearts are in the right place...They're rich guys but they're down to earth...very patriotic, they want to help."

There are also attributes of BENS business model that have facilitated collaboration with public health, including their:

#### Focus on national security and emergency preparedness

BENS' mission-level focus is on national security, and its Business Force program is dedicated to collaboration with state and local governments to advance emergency preparedness. This focus has attracted a membership that includes representatives of very large companies with a sizeable stake in community continuity, as well as people from smaller companies with relevant expertise.

#### Commitment to service

BENS' non-profit and non-partisan status is critical to establishing and maintaining links to governments given potential differences across areas or over time in political leadership. In addition, BENS' firm commitment to being a service-oriented organization is perhaps its single most valued attribute among government partners. BENS zealously guards its reputation in this regard. In a rare instance when a BENS member appeared to government partners as self-promoting, BENS leadership removed that person from the project team.

#### Strategic engagement of senior business and government officials

BENS leadership is strategic in its outreach to government partners at federal, state, and local levels, and BENS staff explicitly identify building personal links as a strategic priority. As part of this approach, BENS is able to leverage the seniority of its members into contacts with senior business and government officials. Business respondents often cited the support of top-level executives in their companies as critical; likewise, government respondents repeatedly cited the governor's support for collaboration with BENS as an essential catalyst for the partnership.

#### Business model

The BENS business model for collaboration includes a)identifying partners' needs and assets, b) developing focused priorities and project concepts that are consistent with its overall mission, and c) initiating projects that can be tested and measurably evaluated. This has been a workable model that has been embraced by both government and business partners.

#### Conceptual link between business and community continuity

As part of its Business Force model, BENS has successfully linked the concept of business continuity planning, an activity that is familiar to business, to the concept of community continuity. Simultaneously, collaboration with BENS has enabled public health leaders to better understand the role of businesses in community continuity as essential to disaster response and recovery.

#### Links to multiple government agencies

The collaborations that BENS has with emergency management, public safety, and homeland security agencies in Georgia have facilitated links with public health. As one public health official noted, "*We all go to each other's meetings*." For example, public health has the lead on SNS dispensing, and officials from other state agencies participate in SNS workgroups. In turn, the Georgia Emergency Management Agency has the lead for projects that address protecting critical infrastructures, linking business partners to the state's emergency operations center, and hurricane preparedness, but public health is represented at these meetings. The credibility that BENS has established and maintains with other agencies *"makes it easier for public health to work with BENS."*

### Benefits of business and public health collaboration

Identifying benefits that have arisen from the collaboration involves two questions: 1) Has the collaboration led to more effective responses to actual emergencies, and 2) Is Georgia better prepared to address a future large-scale emergency?

The most dramatic emergency to occur during the course of the partnership was Hurricane Katrina in August 2005. Although Georgia was not directly hit by the storm, Georgia was the destination for thousands of evacuees from the Gulf coast; and Georgia government agencies and businesses contributed to recovery and relief efforts in directly affected states. The response to Hurricane Katrina did not involve mass dispensing from the SNS in Georgia, the focus of the BENS-public health collaboration, but respondents provided multiple anecdotes describing BENS members' support for the response. As several respondents observed, the director of the BENS Metro Atlanta Region was *"on the phone 24/7" *fielding requests for assistance from government officials and offers of assistance from businesses and making connections to match resources to needs. For example, as the result of business contacts, BENS staff made connections with the airline company that transported evacuees from New Orleans, resulting in better information for public health and healthcare providers about flight arrival times in Georgia and passengers' health status. Other contacts through BENS enabled support for transportation of people and supplies to the Gulf region, assistance in restoring critical telecommunications links, and donations of warehouse space to store and distribute clothing and other donated materials.

Regarding preparedness for a future large-scale public health emergency, all respondents felt that response capacity, particularly in the metropolitan Atlanta area, has been strengthened. Nonetheless, they cautioned that the impact of the collaboration is difficult to quantify, they varied in how they gauged readiness, and they agreed that substantial work remains. Perspectives included the following comments:

"If we had a massive anthrax exposure in the middle of Atlanta tomorrow, we'd probably have a fiasco. But the response would be much more effective than it would have been several years ago, and we are much better positioned to prepare for a future attack than we were several years ago."

"Are we better prepared? Absolutely, but we're not ready. We're halfway through the first quarter."

"We're not done... but if something happened today, we could really help. We have the relationships and a model for corporate involvement. We could get the business commitment. "

Advances in preparedness were also described in more personal terms:

"I can speak straight with him [a colleague in business] in a way that couldn't be done earlier."

"Now we have access to the top folks in business..."

"...People are on one another's speed dials."

"When I call to offer help in a crisis, they [senior government officials] know who I am and they'll take my call."

"*We're learning to think like them and they're learning to think like us*."

More tangible benefits to public health include the engagement of logistics expertise and volunteers from businesses in the July 2005 SNS dispensing exercise and the commitment of businesses to expand the model to all five metropolitan Atlanta health districts. In addition, the success of the partnership has enabled expansion of its agenda to include pandemic influenza planning. For example, the Division of Public Health has involved BENS in encouraging the business community to participate in regional pandemic planning groups throughout the state and in developing procedures for corporate occupational health systems to support home healthcare in the event of a pandemic. Altogether, these activities represent substantial, although un-tallied in dollar terms, investment of private resources into emergency preparedness in Georgia.

### Limits of Business-Public Health Partnerships

Discussion of limitations of partnerships between business and public health fell into the following categories:

#### Limits of volunteerism and *pro bono *engagement

As BENS is being asked to do more by its government partners, the partnership is facing the limits of volunteerism and of *pro bono *member engagement. In this regard, BENS is a "victim of its own success" resulting both from the value that government agencies in Georgia have gained from the partnership and from the ongoing efforts of BENS to promote business-government partnerships. In addition, CDC views the BENS-public health collaboration in Georgia as a model for SNS dispensing that should be emulated by other states, and CDC has enlisted BENS members to consult with businesses and public health officials in other states. As BENS staff or members have observed:

"A few people have donated a lot of time. We need to wrestle with how much time we can ask people to give."

"Potential is unlimited, but we are limited by resources and ability"

"We're being asked to do a lot. It's now more than what we can do. We're being included in everything [regarding emergency preparedness]."

#### Institutional constraints

Limits are also shaped by the lines of accountability and procedures within businesses and governments. Ultimately business managers are accountable to investors or shareholders, and actions must be compatible with companies' long-term interests. For example, a company may endorse the principle of supporting SNS dispensing, but there are limits to how many employees a company may be willing to dismiss from work to participate in a dispensing exercise, which can represent a significant cost. Similarly, public health officials "*live in a political world*" and must act in ways that are politically feasible. Despite the potential value that may be gained from such collaboration, some respondents expressed concern that the partnership may be constrained if the public perceives the SNS dispensing model as a form of favoritism for business.

#### Timing

There are limits to the speed at which business-public health partnerships can take on projects. Efforts to expand the collaboration beyond the Atlanta region within Georgia or to replicate the Georgia experience in other states may not be successful if the scope of projects does not match the status of relationships.

"It has taken five or more years to move from casual 'handshake' relationships to one where people are on one another's speed dials. Relationship building cannot be rushed."

#### Keeping focus on preparedness

Some in public health would like to extend the scope of the partnership to address other health problems, such as promoting workplace obesity prevention or smoking cessation programs. But, there is recognition that pushing in this direction could strain the still-developing relationship with BENS. Noting that attention to emergency preparedness has enabled public health to strengthen infrastructure generally, one public health official cautioned against over-reaching: "*The homeland security dividend only works so far*." While workplace health promotion is certainly a worthy cause, BENS members advised that other business organizations would be a better fit for such efforts.

### Respondent recommendations

Respondents were consistent in recommending that the partnership between BENS and government agencies, including public health, in Georgia be sustained and expanded and that businesses and governments elsewhere initiate similar partnerships. As one public health official noted:

*"I see the public health-business relationship as one of the most useful new relationships for public health that has come onto the horizon"*.

In addition to recommendations to attend to the challenges, facilitators, and limits described above, other recommendations included:

#### Make a start

Cautions against pushing relationships too quickly were offset by a counter-recommendation against moving too slowly.

"You need to make a start, so pick something and get going."

#### Managing growth

Obtaining new funding would enable BENS to push beyond the limits of volunteerism, and both the national office and the Metro Atlanta Region of BENS are exploring potential opportunities for additional funding. Several respondents cautioned that such an expansion could present new challenges for the partnership. Given the long-standing context of BENS' role as an organization that offered volunteer services to governments, initiating contractual arrangements with governments would change the dynamics of BENS-government interactions. For example, expanded funding would allow BENS to pay members whose services are needed beyond the limits of what they can offer *pro bono*. Alternatively, government agencies may be interested in contracting for additional services from BENS members, including those who have previously provided *pro bono *services and who would likely continue to serve as BENS volunteers. This will require defining the line of propriety for members who are both volunteers as well as potential contractors for BENS-related work.

#### Avoiding complacency and overstatement

Despite the achievements to date, substantial work remains to expand the SNS dispensing model to all five metropolitan Atlanta health districts. Beyond Atlanta, where many large companies are based, this model may be less replicable in other, more rural parts of the state. Proponents of the BENS-public health partnership must carefully navigate the boundary between reasonable promotion and overstatement. Public health officials are typically grounded in the scientific tradition, which involves carefully qualifying observations. In contrast, business people are more accustomed to the role of marketing in achieving goals. These differences in perspective can lead to discomfort on both sides of the partnership, another example of the inevitable and ongoing differences in the business and public health cultures.

#### Respecting roles

No respondent challenged the lead role of government in planning or executing emergency response activities, nor did any recommend that government authorities be transferred to businesses. Nonetheless, concern was expressed by some in government about the ability of public health officials to maintain their leadership role. For example, one official noted:

"People in public health tend to have less assertive personalities than people in business. We're dealing with people who run major companies, and they are aggressive and like to take charge. If we're not careful, we could end up handing over control of things that are our responsibility."

"We need to keep in mind, public health is the lead. Public health cannot allow others to be the driving force – partners yes, but not lead. Public health should not turn it over to business to manage. I don't see that happening, but..."

A related recommendation from some public health officials was to assure that governments maintain an option to work directly with businesses that are BENS members, not having to *"go through" *BENS, or to work with other business organizations, without feeling obliged to consider BENS as the sole conduit for business partnerships. Some business respondents anticipated these concerns by emphasizing that BENS should not aim to usurp government authorities or position itself as a gatekeeper for business and public health links, but rather it should continue its role in supporting government programs and facilitating links. As one BENS staff member commented, *"We don't have all the answers, there's room for others." *Another dimension of respecting government and business roles was expressed by a company executive in the following way:

"It is important for all parties involved to understand the 'value proposition' for the other parties. If we are not creating value for other members, the team will not survive. If we can to that, we can sustain our effort."

## Discussion

Collaboration between BENS and public health agencies in Georgia has produced a model for mass dispensing of SNS pharmaceuticals that involves integration of public and private resources and capabilities. The partners have subjected their model to "proof-of-concept" testing and are working to extend it to multiple public health districts in metropolitan Atlanta. Five key lessons arising from our examination of the partnership are:

### The partnership arose and flourished because BENS leadership was strategic in its outreach to government officials and because government officials were willing to entertain a new type of relationship with businesses

BENS is a non-profit entity whose members include people and companies from the for-profit sector. As such, BENS has carefully defined its mission and methods in ways that are sensitive to the historical reticence of their business colleagues and government officials to engage with one another. This approach has facilitated a focus on shared objectives and a process that has enabled participants to overcome obstacles to collaboration.

### The partnership readily garnered support from people in business and public health because mass SNS dispensing represents a clear example of the potential benefits of government-business collaboration

Rapid, mass dispensing of post-exposure prophylactic medications to the residents of a large urban area is obviously a task that government alone would be unable to manage successfully, and it is equally obvious that the consequences of failure could be devastating. Even if businesses were able to provide prophylactic medications to their employees, businesses could not survive if customers and those who maintain necessary infrastructures were unprotected because governments were overwhelmed. At the same time, it is evident that public health agencies and businesses have complementary skills and assets that, if combined, would increase opportunities for successful mass dispensing.

### There are multiple and complex challenges to collaboration, including some that can be managed but probably not eliminated and others that may be resolved through ongoing efforts

While our investigation identified multiple challenges to collaboration, the business and public health leaders responsible for forging the partnership in Georgia viewed their ability to recognize and confront these challenges as an important accomplishment and not as a deterrent to advancing the partnership. In particular, the cultural divide between people in business and government has been narrowed, and personal trust and respect have grown through collaboration. This achievement, however, cannot be taken for granted as the partnership grows and new participants from businesses and governments become engaged. Many of the challenges we identified emerged as the partnership addressed progressively more complex tasks, from preparing an informational pamphlet to designing and testing an SNS dispensing model, extending that model to new sites, and adding pandemic influenza planning to the partnership agenda. This process surfaced concerns about the limits of volunteer engagement and the danger of complacency, which will require ongoing attention, and about liability and confidentiality of sensitive information, which may be amenable to legislation. For example, most state Good Samaritan laws provide liability protection to individuals but do not extend that protection to businesses or non-profit organizations that assist in an emergency response; moreover, these laws typically come into play once disasters occur but do not apply during pre-event drills or exercises (personal communication, November 8, 2006, Gene Matthews, JD, former CDC General Counsel and Senior Fellow, University of North Carolina School of Public Health). These limits of Good Samaritan laws may discourage some businesses from engaging in public health emergency planning and exercises, and efforts to describe and characterize their limits can provide the foundation for efforts to seek legislative or policy changes.

### The BENS partnership model resonated with business and public health colleagues alike

This model is based on identifying shared objectives, identifying respective assets and liabilities, and implementing manageable and measurable projects that fit within both government and business missions. While this may sound elementary, maintaining this focus has required ongoing effort.

### The concept of community continuity links principles of business continuity and public health

The importance of business continuity planning is widely accepted among business leaders. Public health officials are accustomed to taking a community-level perspective in seeking to protect and promote health. The link between these concepts is that a healthy population (e.g., healthy employees, customers, suppliers) is essential to business survival during and following a public health crisis, and viable businesses are essential to provide jobs, essential goods and services, and a sense of economic well-being necessary to support health[17]. Calls for a comprehensive approach to disaster planning, response, and recovery emphasize the importance of the economic impacts of disasters on physical and mental health [18,19]. Businesses can also play a key role in supporting the recovery of communities from an epidemic or disaster. This was illustrated in 2003 following the SARS epidemic in Toronto. Conventions and tourism – two of the city's key industries – suffered when organizations cancelled conventions and tourists turned away. Responding to this threat to the economic health of Toronto, businesses that would normally be competitors, such as airline, rail, and bus companies, collaborated to foster the return of conventions and tourists [20].

Both domestically and internationally, there is growing interest in partnerships between business and governments for addressing a spectrum of public health threats, including obesity, tobacco use, bioterrorism [21], pandemic influenza [22], and HIV and other infectious diseases of global concern [23]. Yet, as Reich commented in summarizing a Harvard/Global Health Council conference on public-private partnerships for public health in 2000,

"We know little about the conditions when partnerships succeed. Partnerships can produce innovative strategies and positive consequences for well-defined public health goals, and they can create powerful mechanisms for addressing difficult problems by leveraging the ideas, resources, and expertise of different partners. At the same time, the rules of the game for public-private partnerships are fluid and ambiguous" [23].

As noted by others at the same conference, "A chief factor encouraging these partnerships is that neither side can achieve its specific goals alone; collaboration is unavoidable," and "cross-sector partnerships do not happen; they are built" [24,25]. While these observations arose from an examination of public-private partnerships in public health outside the United States, they are remarkably germane to our observations regarding collaborations between public health and businesses in Georgia.

The term "public-private partnership" itself, although widely used, is vague and embraces a spectrum of activities including outsourcing government functions to private companies, engagement of private consultants to advise governments, the work of philanthropies, and active collaborations [23,26]. The BENS-public health project is an active collaboration involving senior and operations-level staff from businesses and public health agencies in planning, developing, implementing, and testing an SNS dispensing model and, more recently, in supporting pandemic influenza planning. As such, this is a collaboration that was deliberately built through long-term, sustained efforts of the partners. Building such relationships requires patience and time. People from business must recognize that governments are usually less nimble than private companies, as learned by Arnold Schwarzenegger, Governor of California, in describing his personal transition from business to government, "You have to have a bit more patience. Coming from the private sector, not having been involved in government, I wanted to do things as quickly as in the private sector, and that doesn't happen" [27].

In building partnerships with business, people from governments cannot set aside their responsibilities for regulation or addressing health threats when they arise from business activities or commercial products, but, at the least, they must learn to view businesses through another lens as collaborators for the public good. This need did not arise with the threat of bioterrorism. For example, in 1992, CDC launched a "Business Responds to AIDS" program, including outreach to 35,000 corporations to solicit their engagement in addressing the threat of HIV, which affects communities at large and businesses [28].

The ground rules for public-private partnerships in public health are in flux. As noted by Hershey at the Second Annual Partnership Conference on Public Health Law, convened in 2003 by CDC,

"A local public health agency and its public health officer are faced with difficult issues regarding public-private partnerships. These include: 1)Congruency of mission and goals – Are the missions and goals of the partnership consistent with that of the local public health agency?; 2) Conflicts of Interest – Is there a perceived or real conflict of interest in the partnership?; 3) Conflicts of Obligation/Accountability – To whom and to what are local public health agencies accountable?; 4) Balancing Ethical Rules and Values – What ethical rules and values are local public health agencies balancing? Who's [*sic*] rules and values should they protect? What is the role of the public health professional?; and 5) Allocation Issues – Who decides what gets funded?" [29]

These questions may become especially relevant for the BENS-public health partnership in Georgia. The model of collaboration that has been developed for SNS dispensing in Georgia has not been subject to wide-scale public scrutiny, and, in the absence of an actual emergency requiring SNS dispensing, may never come to that level of public attention. Whether the proposed model is welcomed by the public and media or viewed as an example of government favoritism to business remains to be seen. If it comes to widespread public attention, this question will be likely resolved in the political arena. In addition, as CDC seeks support from BENS to replicate the Georgia SNS dispensing model in other states and as public health in Georgia seeks to expand its portfolio of projects with BENS, it may be necessary to contract with BENS or individual BENS members if the demand for services exceeds their capacity to work *pro bono*. Possible mixing of volunteer work with activities funded through contracts or other arrangements will change relationships with government agencies, raising potentially difficult ethical and procedural questions for BENS, its members who are both volunteers and potential paid consultants, and governments.

## Conclusion

The partnership between businesses and public health agencies in Georgia to develop SNS dispensing capacity is based on mutual trust and shared objectives: protecting public health in the event of a bioterrorist attack or other major infectious disease emergency and, by extension, contributing to community cohesion in the wake of a large-scale disaster. Achieving this objective fits directly with the mission of public health and recognizes the interaction between business and community continuity. The partnership also rests on the realization that combining government and business resources increases opportunities for successful emergency preparation and response. Establishing and maintaining this partnership has required that participants acknowledge and address multiple challenges, including cultural differences and operational constraints, such as concerns about the confidentiality of shared information, liability, and the limits of volunteerism. Through collaborative engagement in progressively more complex projects, the partners have made considerable progress in overcoming these obstacles. As a result of the partnership, essential relationships have been established, substantial private resources and capabilities have been engaged in government preparedness programs, and a model for collaborative SNS mass dispensing has been developed, tested, and slated for expansion. The lessons learned from this collaboration in Georgia should be considered by other government and business leaders seeking to develop similar partnerships.

## Competing interests

The authors declare that they have no competing financial interests. JB serves as a consultant to the Georgia Division of Public Health, and RB has been an active proponent of public health and business partnerships in metropolitan Atlanta, including serving as the co-chair of the Business-Public Health Partnership Roundtable, organized under the auspices of the Sam Nunn School of International Affairs of the Georgia Institute of Technology

## Authors' contributions

JB was the lead investigator and lead author of this report and was involved in all aspects of project development, study design, and implementation, including conducting interviews, analyzing interview notes, and interpreting the study results. EW contributed to study design and implementation, including conducting interviews, analysis and interpretation of findings, and report preparation. RB provided expertise in public-private partnerships and emergency preparedness, and she contributed to the development of study objectives, study design, interpretation of results, and report preparation. All authors read and approved the final manuscript.

## Pre-publication history

The pre-publication history for this paper can be accessed here:



## Supplementary Material

Additional file 1 **Partnership history**. This document provides a brief history of the partnership between the Metro Atlanta Region of the Business Executives for National Security and the Division of Public Health of the Georgia Department of Human Resources. Click here for file

Additional file 2 **Study participants**. This document provides the names, titles, and affiliations of the people interviewed in the case study investigation. Click here for file

Additional file 3 **Interview questions**. This document lists the questions that the interviewers asked of the study participants. Click here for file

## References

[B1] United States General Accounting Office Bioterrorism, Public Health Response to Anthrax Incidents of 2001. GAO-04-152.

[B2] United States Government Accountability Office Bioterrorism, Information on Jurisdictions' Expenditure and Reported Obligation of Program Funds. GAO-05-239.

[B3] Health Resources and Services Administration National Bioterrorism Hospital Preparedness Program. http://www.hrsa.gov/bioterrorism/.

[B4] Centers for Disease Control and Prevention Cooperative Agreement Guidance for Public Health Emergency Preparedness. http://www.bt.cdc.gov/planning/guidance05.

[B5] United States Department of Health and Human Services HHS Announces $100 Million to Accelerate State and Local Pandemic Influenza Preparedness Efforts (News Release). http://www.hhs.gov/news/press/2006pres/20060112.html.

[B6] Levenson E, Leonard D (2005). Crisis management: The only lifeline was Wal-Mart. Fortune.

[B7] Hafner K, Deutsch CH, Fabrikant G, Ruethling G, Whitmire K Storm and Crisis: The Helping Hands; When Good Will Is Also Good Business. New York Times.

[B8] National Business Group on Health Corporate America and Emergency Preparedness: Employer Perspectives on Working with the Public Health Sector. Terrorism and Public Health Emergency Preparedness Initiative Analysis Paper. http://www.businessgrouphealth.org/prevention/employertoolkit/corpamer_employerperspective.pdf.

[B9] National Business Group on Health Corporate America and Emergency Preparedness: Public Health Perspectives on Working with the Business Sector. Terrorism and Public Health Emergency Preparedness Initiative Analysis Paper. http://www.businessgrouphealth.org/prevention/employertoolkit/corpamer_publichealth.pdf.

[B10] Centers for Disease Control and Prevention Strategic National Stockpile. http://www.bt.cdc.gov/stockpile/.

[B11] Centers for Disease Control and Prevention Emergency Preparedness & Response: City Readiness Imitative. http://www.bt.cdc.gov/cri/.

[B12] Business Executives for National Security Mission Statement. http://www.bens.org/about.html.

[B13] Business Executives for National Security National Business Force. http://www.bensbusinessforce.org/.

[B14] Internal Revenue Service Publication 557 (3/2005), Tax-Exempt Status for Your Organization Section 501(c)(3) Organizations.

[B15] Business Executives for National Security Metro Atlanta Region Homeland Security Advisory Group (2004). Getting Ready: Company Primer on Preparedness and Response Planning for Terrorist and Bioterrorist Attacks.

[B16] Brinkerhoff RO (2003). The Success Case Method: Find Out Quickly What's Working and What's Not.

[B17] Patterson J (1999). A review of the literature and programs on local recovery from disaster (Working Paper #102). Natural Hazards Research and Applications Information Center, Institute of Behavioral Science, University of Colorado, issued by Public Entity Risk Institute.

[B18] Barnett DJ, Balicer RD, Blodgett D, Fews AL, Parker CL, Links JM (2005). The application of the Haddon matrix to public health readiness and response planning. Environmental Health Perspectives.

[B19] Weisler RH, Barbee JG, Townsend MH (2006). Mental health and recovery in the Gulf coast after Hurricanes Katrina and Rita. JAMA.

[B20] Matthews G The Public Private Response to Sudden Disease Outbreak: Convening key stakeholders in the Unites States and Canada to re-examine the outbreak of SARS in Toronto as a basis for strategic planning by both government agencies and the corporate sector to more effectively meet the challenges of future incidents worldwide. Final report prepared for the Alfred P. Sloan Foundation. Institute for Public Health Law, CDC Foundation.

[B21] Simon PA, Felding JE (2006). Public health and business: a partnership that makes cents. Health Affairs.

[B22] Centers for Disease Control and Prevention State and Local Pandemic Influenza Planning Checklist, Community Preparedness Leadership and Networking. http://www.pandemicflu.gov/plan/statelocalchecklist.html#preparedness.

[B23] Reich MR, Reich MR (2002). Public-private partnerships for public health. Public-Private Partnerships for Public Health.

[B24] Reich MR (2000). Public-private partnerships for public health. Nature Medicine.

[B25] Barrett D, Austin J, McCarthy S, Reich MR (2002). Cross-sector collaboration: lessons from the International Trachoma Initiative. Public-Private Partnerships for Public Health.

[B26] Gerrard MB (2001). Public-private partnerships. Finance Development.

[B27] Steinhauer J The 2006 election: a major race; Schwarzenegger Voices New Confidence. New York Times.

[B28] Centers for Disease Control and Prevention (1993). Effectiveness in disease and injury prevention Business Responds to AIDS Program – December 1992–February 1993. Morbid Mortal Weekly Rept.

[B29] Reich MR, Hershey JH, Hardy GE, Childress JF, Bernheim RG (2003). Workshop on public health law and ethics I & II: the challenge of public/private partnerships (PPPs). J Law Medicine Ethics.

