# Supplementary material for: Business and public health collaboration for emergency preparedness in Georgia: a case study
Source: BMC Public Health. 2006 Nov 20;6:285. doi: 10.1186/1471-2458-6-285 (PMC1676007; doi:10.1186/1471-2458-6-285)
Supplement: Additional file 1 — Partnership history. This document provides a brief history of the partnership between the Metro Atlanta Region of the Business Executives for National Security and the Division of Public Health of the Georgia Department of Human Resources. [file 1471-2458-6-285-S1.doc]

**Additional file 1 – Brief history of the partnership between the Metro Atlanta Region of the Business Executives for National Security and the Division of Public Health of the Georgia Department of Human Resources: Business and public health collaboration for emergency preparedness in Georgia: a case study**

As a national, nonprofit, nonpartisan organization, the mission of the Business Executives for National Security (BENS) is to provide a “channel through which senior business executives can help enhance the nation's security” (See: Business Executives for National Security [<http://www.bens.org/about.html>]). The direction of BENS has been shaped by recent historical events. These include the collapse of the Soviet Union and the end of the cold war, the emergence of state-sponsored and non-state terrorism as a threat to the United States, the events of September 11, 2001, the subsequent anthrax attacks, the spread of avian influenza and the growing visibility of a possible influenza pandemic, Hurricane Katrina, and the ongoing threat of catastrophic natural, unintentional, or intentional events.

Since 2000, the Metro Atlanta Region of BENS has cultivated a multi-faceted partnership with government agencies in Georgia, including the Georgia Emergency Management Agency, the Office of Homeland Security, the Georgia Department of Defense (National Guard), the Office of Public Safety, the Office of the Governor, and the state and district-level offices of the Division of Public Health of the Department of Human Resources. Additionally, in part because of its Atlanta location, BENS Metro Atlanta has established a strong partnership with CDC. These collaborations have resulted in a mix of projects geared to strengthening the state’s capacity to detect, respond to, or recover from a bioterrorist attack or other large-scale epidemic or disaster.

After September 11, 2001, BENS initiated a Business Force program in several states that broadly engages business leaders and companies in collaborative projects, primarily with state and local governments (See BENS Business Force [<http://www.bensbusinessforce.org/>]). The Business Force program represents an evolution that some BENS members describe as an expansion from “classic" or "traditional" BENS to “expanded BENS” activities. “Classic BENS” focused (and continues to focus) on bringing best business practices and models to the "business" of national security. The goal is to reduce support costs and use the savings to support higher priority core competencies. In these activities, BENS members have served mainly as facilitators and consultants to federal and state agencies, and collaboration has typically focused on BENS efforts to assist government managers in improving both efficiency and effectiveness by applying insights from business management practices. Many BENS members have had prior careers in the military, and they value the opportunity that BENS membership provides to “continue to serve” and to maintain or develop links with active-duty military officers or like-minded former officers now employed in public safety agencies or business.

The evolution to “expanded BENS” was shaped by a realization that for certain large-scale emergencies, whether arising from acts of terrorism, natural disasters, or disease epidemics, neither governments nor businesses alone can sufficiently prepare for, respond to, or recover from such events. This awareness was accompanied by a growing recognition that governments and business each have resources and capabilities that, if combined, could enhance emergency preparedness to the mutual benefit of both and more broadly for the benefit of communities. For businesses, this included recognition that government agencies have unique expertise and information resources, especially with regard to dimensions of emergency preparedness, such as infectious diseases, that are less familiar to businesses. This includes knowledge about biological threats, access to information from multiple levels of government in crisis situations, and the skills and authorities essential for responding to biological and other public health emergencies. In Georgia, “expanded BENS” activities have been organized under the BENS Georgia Business Force, which is the platform for broader involvement of companies in collaborative projects with state and local governments. The Business Forces involves BENS member companies, their senior company officers as well as company operations and security executives. Although BENS Metro Atlanta has worked with Georgia’s Division of Public Health for years, the establishment of the BENS Georgia Business Force in 2003 led directly to much greater engagement with public health.

The partnership between BENS and public health agencies in Georgia had its antecedents in outreach that the BENS leadership in Georgia made to the Centers for Disease Control and Prevention (CDC), a federal agency headquartered in Atlanta, and to the state’s Director of Homeland Security. Links to CDC were established before the terrorist attacks of 2001. Conrad "Connie" Busch, Director of the Metro Atlanta BENS chapter, and other BENS leaders engaged senior officials at CDC, including Kathy Cahill, CDC Associate Director for Policy and Planning, and Joe Henderson, Director of CDC's Office of Terrorism and Emergency Response, in planning and executing a BENS-sponsored bioterrorism tabletop exercise, held in July 2000. Following this initial work with CDC, BENS sought greater engagement with public health officials in Georgia, and Ms. Cahill referred BENS to Dr. Scott Wetterhall, a senior CDC epidemiologist assigned to the DeKalb County Board of Health. DeKalb County encompasses the eastern part of the City of Atlanta and the adjacent City of Decatur. It is one of five public health districts that comprise the metropolitan Atlanta area (See Georgia Division of Public Health [<http://health.state.ga.us/regional/index.asp>]), and it has a population of 676,000 (2004 estimate, Source: Georgia Division of Public Health [<http://oasis.state.ga.us/>]). This was a logical step since DeKalb County was one of three local health departments in the country funded by CDC as "Advanced Practice Centers" for emergency preparedness (see: <http://www.dekalbhealth.net/cphp/about-cphp.htm>), and DeKalb's area of emphasis as a Center was partnership development. This led to conversations with DeKalb public health officials later in 2000 and early 2001 and the development of a BENS Metro Atlanta-DeKalb County Public Health homeland security partnership, which identified the need to inform business about bioterrorism preparedness as a priority.

In January 2001, BENS leaders offered the organization's services to the state’s Commissioner of Public Safety. In May 2002, BENS members began attending state Homeland Security Working Group (HSWG) meetings regularly at the suggestion of the Commissioner of Public Safety. Following the Group of Eight (G8) international summit meeting in June 2004, held on Sea Island, Georgia, the HSWG was not reconvened and, at the invitation of the state Director of Homeland Security, BENS members attended meetings of the state’s Homeland Security Task Force. Members of the Task Force include the directors of state agencies responsible for planning and executing emergency response activities, including public health, (see: <http://www.gema.state.ga.us/ohsgemaweb.nsf/>). Through its engagement with the Homeland Security Task Force, BENS gained credibility with Task Force members, who comprised the leaders of the state’s public safety, emergency management, and public health agencies. This led to a meeting of the BENS national President and CEO and Georgia BENS leaders with the Georgia Director of Homeland Security and Governor in October 2003. At this meeting, the governor and BENS President and CEO formally agreed to establish a BENS-Georgia homeland security partnership. As a result, the Governor and his Homeland Security Director actively encouraged state agencies engaged in emergency preparedness to collaborate with BENS.

Dr. Kathleen Toomey, the state’s health officer and Director of the Division of Public Health of the Georgia Department of Human Resources, served as a member of the Homeland Security Task Force and was introduced to BENS leaders through their involvement with the Working Group. As part of their engagement with the Homeland Security Task Force, BENS members worked with the state’s Division of Public Health, the Office of Homeland Security, the DeKalb Board of Health, and CDC to write *Getting Ready: A Company Primer on Preparedness and Response Planning for Terrorist and Bioterrorist Attacks* (<http://www.bens.org/images/GettingReady_042304.pdf>). Published in April 2004 as a collaboration with state and DeKalb public health officials, the primer featured a forward by the Governor and included contact information for key state agencies, including each of the state’s 19 public health districts (since that time, two districts were coalesced, and there are currently 18 public health districts in Georgia).

The governor's endorsement of the partnership in 2003 was a potent incentive for public health to expand its links to BENS. The next phase of engagement between BENS and public health focused on assessing the state's capacity to respond to a large-scale bioterrorist attack. State emergency preparedness staff identified the daunting task of developing and exercising systems for distributing medications from the Strategic National Stockpile (SNS) as an obvious priority. In particular, the task of identifying, organizing, training, and maintaining large numbers of volunteers who would support such an effort was viewed as particularly challenging. Based on relationships established via Homeland Security Task Force and the DeKalb Board of Health, the state Division of Public Health organized a meeting with BENS leaders, the DeKalb Board of Health, and the Cobb-Douglas Health District (another of the five core metro Atlanta health districts) to discuss possible collaboration in developing SNS distribution systems. These discussions followed what has become a model for BENS Business Force collaborations with government and were based on answering the following questions:

- What needs to be accomplished?
- What are the assets that each party brings to the task (or series of tasks)?
- What are the gaps or unmet capacities that each party faces in accomplishing the task(s)?
- How can the partners combine resources and assets in a mutually beneficial way to meet task objectives?
- Which specific task activity should be targeted for initial development and implementation?
- How can objectives and performance yardsticks be defined in a way that allows proposed activities to be tested and rigorously evaluated?

Reflecting federal mandates under the Cities Readiness Initiative (CRI, see URL <http://www.bt.cdc.gov/cri/>), the scenario that served as the basis for these conversations was a large-scale exposure to aerosolized anthrax in the metro Atlanta area and subsequent mass distribution of post-exposure prophylaxis using medications supplied from the SNS. The necessary chain of events in this process would include receiving SNS supplies, “breaking down” mass shipments, distributing medications to clinic sites, and administering the medications to large numbers of people at these sites. Following a discussion based on the above model, public health officials and BENS leaders agreed to focus on developing and testing procedures for mass dispensing clinics. This decision was based on the need for large numbers of volunteers to support clinics and public health's need for technical assistance in addressing the complex logistic challenges involved in implementing such clinics. Although work began on the project in late 2003, it was temporarily suspended when state public health and homeland security officials were required to redirect their attention to preparing for the international G8 summit conference on Sea Island, Georgia in June 2004.

The collaboration resumed upon completion of the G8 conference and culminated in the Atlanta Metropolitan Biological Emergency Response (AMBER) exercise of mass distribution clinics in July 2005. Exercises were conducted in all five of the core metropolitan Atlanta public health districts and involved collaboration with BENS in two districts, DeKalb and Cobb-Douglas. In these two districts, BENS volunteers worked closely with state and local public health officials to design clinic procedures and implement the exercise, and approximately 1,200 volunteers recruited by BENS served as mock patients, dispensing site workers, and exercise evaluators. At one site in each, the clinics were managed by public health officials in school facilities. At a third site in the Cobb-Douglas district, the exercise was conducted at a BENS-member company facility, and company volunteers played a major role in managing the clinic. The onsite and after-action evaluations led to substantial changes in thinking about how point-of-dispensing sites should be managed, including changes in recommended procedures for providing information to people attending the clinic, selecting buildings to serve as clinic sites, and for providing security. Subsequent deliberations also brought into focus a model of business-public health collaboration in SNS distribution following an anthrax attack that is characterized by the following attributes and benefits:

- As an adjunct to distribution sites managed by local public health departments, large companies would take responsibility for managing a distribution clinic at a company facility. This facility would distribute SNS materials primarily to company employees and their family members. The company would be responsible for recruiting and maintaining volunteers to staff the clinic, developing procedures to provide pre-training and just-in-time training for volunteers, and managing the clinic based on logistic procedures developed through the BENS-public health partnership and on healthcare standards set by public health.
- Upon completion of dispensing medications at the company facility, company volunteers would serve in public health dispensing sites and support government efforts to distribute SNS supplies.
- Companies would benefit by having assured access to SNS supplies for their employees and families, key for business continuity. Businesses would also benefit from the community-wide benefits of preventing widespread illness and attendant benefits associated with maintenance of community cohesion.
- Public health would benefit because large numbers of people would receive SNS supplies from their employers, reducing the strain on sites managed by public health departments. Public health would also benefit from the expertise and insights of business logistic and management experts and from the volunteers that would be available from businesses to support public health distribution sites.
- Communities benefit because businesses remain in business—companies employ workers, provide essential goods and services to the community, and support local economies.
- Government benefits because key companies “stay in business” and provide essential goods, services and jobs necessary for the community. If companies are not in business, then government must provide selected goods and services to make up the deficit.

Following the AMBER exercise, BENS leaders and public health officials planned to extend exercises of this SNS distribution model to all five core metro-area health districts. This was initially scheduled to be held in the fall of 2006. However, the demands on public health departments for pandemic influenza planning forced postponement of this planned model extension to early 2007 and simultaneously prompted opening of a new line of collaboration to implement systems to support home care of people suffering from influenza.

As work with SNS distribution and dispensing was ongoing with public health, BENS remained engaged in collaborations with public safety and emergency management agencies in Georgia. Projects or activities involved supporting security planning in for the G8 conference in 2004 and contributing to federal, inter-state, and Georgia-specific responses to Hurricane Katrina in 2005. Ongoing collaborations include developing systems to identify and protect privately owned “critical infrastructure” resources in Georgia. A new effort is underway to develop procedures to bring the considerable assets of the business community to help government respond to natural or people-made catastrophic events. In order to develop the processes for an organized business community response, businesses have been linked to the state’s emergency operations center in advance of the 2006 hurricane season. The Business Operations Center (BOC) is fully integrated with the state’s emergency operations center. Business representatives from each critical infrastructure area, e.g., power generation, will have a seat at the BOC and formal communication links will be established between the BOC and state’s emergency operations center as well as between the BOC and businesses.

**Acknowledgement**

Conrad "Connie" Busch, Jr., APR, Director, Metro Atlanta Region, Business Executives for National Security provided information on the history of BENS in Georgia for this report.
