# Supplementary material for: Business and public health collaboration for emergency preparedness in Georgia: a case study
Source: BMC Public Health. 2006 Nov 20;6:285. doi: 10.1186/1471-2458-6-285 (PMC1676007; doi:10.1186/1471-2458-6-285)
Supplement: Additional file 2 — Study participants. This document provides the names, titles, and affiliations of the people interviewed in the case study investigation. [file 1471-2458-6-285-S2.doc]

**Additional file 2 – People interviewed for the case study: Business and public health collaboration for emergency preparedness in Georgia: a case study**

Business Executives for National Security, Metro Atlanta Region Office

Conrad H. Busch, Jr., Director

John H. Turner, III, Director and Program Manager, Georgia Business Force

Business Executives for National Security, National Office, Washington, DC

Ern Blackwelder, Senior Vice President, BENS Business Force

Business Executives for National Security, Metro Atlanta Chapter and Georgia Business Force, Members

Anthony Begando, Chief Executive Officer. Tenon Consulting Solutions

William F. Brumund, PE, Principal, Golder Associates Inc.

David Crow, Senior Partner, Accenture

Brian Johnstone, Executive Director, Government Affairs Aeronautics, Lockheed Martin Aeronautics Company

Charles J. Lanthram, Vice President for Security and Business Control, BellSouth Corporation

Chris Melton, The White Oak Group

Mark Wood, MD, Lockheed Martin Aeronautics Company

Division of Public Health, Georgia Department of Human Resources

Stuart Brown, MD, Director

Patrick O'Neal, MD, Medical Director, Office Emergency Medical Services, Trauma, and Emergency Preparedness

Kathleen Toomey, MD, MPH, Former Director

Calita S. Richards, Pharm.D., MPH, State Strategic National Stockpile & CHEMPACK Coordinator, Office Emergency Medical Services, Trauma, and Emergency Preparedness

Lee Smith, Bioterrorism Preparedness Coordinator, Office Emergency Medical Services, Trauma, and Emergency Preparedness

Cobb-Douglas Public Health District Office, Marietta, GA

Pam Blackwell, RN, Director, Center for Emergency Preparedness & Response

Alpha Bryan, MD, Director

Leticia Mathis, Emergency Preparedness Specialist

DeKalb County Board of Health

William Glisson, Former Emergency Preparedness Coordinator

Centers for Disease Control and Prevention

Toby Merlin, MD, Acting Director, Division of Public & Private Partnerships, National Center for Health Marketing

Scott F. Wetterhall, MD, MPH, Senior Program Director, Health Security & Systems Research, Research Triange Institute International, Former CDC medical epidemiologist assigned to the DeKalb Board of Health Center for Public Health Preparedness

Georgia Office of Homeland Security and Department of Public Safety

Donna Burns, Executive Asst. to Director of Homeland Security, and Director, Public Affairs, Office of Homeland Security

Colonel Bill Hitchens, Commissioner, Department of Public Safety

Micheal Sherberger, Retiered, Former Director Georgia Office of Homeland Security

Academia

William E. Hoehn, Jr., PhD, Visiting Professor and Coca-Cola Eminent Practitioner, The Sam Nunn School of International Affairs, Georgia Institute of Technology

Ruth L. Berkelman, MD, Director, Center for Public Health Preparedness & Research, Rollins School of Public Health, Emory University
