# Supplementary material for: Business and public health collaboration for emergency preparedness in Georgia: a case study
Source: BMC Public Health. 2006 Nov 20;6:285. doi: 10.1186/1471-2458-6-285 (PMC1676007; doi:10.1186/1471-2458-6-285)
Supplement: Additional file 3 — Interview questions. This document lists the questions that the interviewers asked of the study participants. [file 1471-2458-6-285-S3.doc]

**Additional file 3 – Interview questions: Business and public health collaboration for emergency preparedness in Georgia: a case study**

1. Please describe your role in the BENS-public health partnership in Georgia.
   1. How long have you been involved?
   2. Which activities or projects have you been involved in?)
2. What motivated you to get involved in this partnership?
3. For those involved in establishing the partnership:
   1. How were the objectives and priorities for the partnership established?
   2. How were these objectives and priorities translated into specific projects?
4. For those not involved in establishing the partnership but involved in specific projects:
   1. How were the objectives and priorities for the project established?
   2. How were these objectives and priorities translated into specific activities?
5. From your perspective, what is the evidence that the partnership and its projects have led to improved preparedness for public health emergencies in the metropolitan Atlanta area? (Probes: For example, has the partnership improved bioterrorism preparedness, planning for pandemic influenza, the infrastructure for sharing information between business leaders and public health officials, or the response to actual events, such as the influx of evacuees following Hurricane Katrina?
6. What were or are the hurdles or barriers to the partnership, including historical and persistent concerns?
7. How were these barriers overcome, or how are they being addressed?
8. What situations, circumstances, or events made the collaboration easier, and how were these opportunities exploited?
9. What are realistic expectations for partnerships between business organizations like BENS and public health agencies?
10. What recommendations can be offered to improve the partnership?
11. What recommendations can be made to others who are considering the development of a business-public health partnership?
    1. Recommendations for business leaders
    2. Recommendations for public health leaders
